# Regeneration Limitations of *Hippophae rhamnoides* Population After Successfully Encroached on the Qinghai‐Tibetan Plateau

**DOI:** 10.1002/ece3.70684

**Published:** 2024-12-22

**Authors:** Baoli Fan, Pengfei Gao, Tingting Tian, Jinhua Jiang, Nana Ding, Yongkuan Wan, Miaojun Ma, Kun Sun

**Affiliations:** ^1^ College of Life Science Northwest Normal University Lanzhou China; ^2^ Key Laboratory of Eco‐Functional Polymer Materials of the Ministry of Education Lanzhou China; ^3^ College of Ecology Lanzhou University Lanzhou China

**Keywords:** population regeneration, seed and bud bank, seed germination, seedling establishment, water transport channels

## Abstract

Shrub encroachment can alter the structure and function of grassland ecosystems, leading to their degradation. Therefore, population regeneration dynamics after shrub encroachment on the influence of grassland should not be ignored. *H. rhamnoides* , as a pioneer species, has significantly encroached with large areas on the Qinghai‐Tibetan Plateau (QTP) due to climate change and over‐grazing. However, few studies have focused on the dynamics of population regeneration following successful encroachment. Therefore, we studied *H. rhamnoides* natural population in the alpine grasslands, investigating population regeneration pattern, seed, bud production and storage, and limitation imposed by microhabitats (soil, light and feeding). Our aim was to explore population regeneration strategies and identify key limiting factors for population regeneration after successful encroachment. Our findings revealed several key points: (i) *H. rhamnoides* entered the alpine grassland by relying on seeds, it would seize resources by low‐cost clonal reproduction, then increase sexual reproduction to improve genetic diversity. (ii) The production and storage of seeds and buds was sufficient, seed vigor was high, seed emergence rate was higher due to mechanical restriction of hard seed coat was weakened by the water transport channels in the palisade layer, and formation of seedlings was less restricted. (iii) *H. rhamnoides* population regeneration was mainly limited by microhabitats light and feeding. However, light and feeding significantly affected seedlings photosynthesis and carbon storage, their interaction significantly reduced the seedlings survival, and further restricted population regeneration. The results can provide theoretical basis for the restoration and management of alpine grassland degradation caused by shrub encroachment.

## 1 Introduction

Shrub encroachment is a phenomenon that occurs in grassland ecosystems where shrubs or woody species increase in density, cover and biomass, leading to a reduction in herbaceous cover. This can significantly impact plant growth, distribution, soil properties as well as the structure and function of grassland ecosystems to varying degrees, resulting in declining ecosystem function and grassland degradation (Van Auken 2009; Báez and Collins 2008; Zhou et al. 2019). QTP also known as “the third pole in the world”, is a unique natural environment hosting the largest and most diverse range of alpine grassland, and changes in its structure and function can be used as warning signal of environment changes (Wang et al. 2020). One significant change contributing to the severe degradation of these alpine grasslands is shrub encroachment, which has markedly compromised its ecological security barrier function (Báez and Collins 2008). This degradation is directly linked to climate change and overgrazing, and which is an important ecological problem facing the global grassland ecosystem (Liu et al. 2023; Shi et al. 2023). The extensive encroachment is driven by their high sexual and clonal reproduction capacity, while the dynamics of population regeneration following encroachment into alpine grasslands remain unclear.

Plant population regeneration is a dynamic process whereby seedlings or ramets emerge and survive to mature, eventually replenishing the population (Harper 1977; Khaine et al. 2018). Many perennials reproduce both sexually and clonally, balancing between these strategies due to resource limitations (Yang et al. 2009; Pausas and Keeley 2014). Regeneration via seedlings enhances population genetic diversity, offering resilience to long‐term changes, whereas ramets serve as effective short‐distance reproduction (Pérez‐Harguindeguy et al. 2013). When sexual reproduction via seedlings fail to maintain populations, replenishment through clonal reproduction ensures population persistence and expand them (Ott, Klimešová, and Hartnett 2019). However, plants face limitations throughout their life cycle, including seed production, dispersal, germination and seedling establishment (Nathan and Muller‐Landau 2000). Formation of ramets or seed production, dispersal from the parent plant, followed by germination at suitable sites and subsequent seedling establishment are constrained by various biotic and abiotic factors (Huang et al. 2003; Li et al. 2011; Yue et al. 2019). Adverse conditions imposed by these factors often lead to increased seedling mortality (Moles and Westoby 2004).

Recruitment limitation is a critical ecological process affecting population dynamics, species composition, abundance and diversity at the local community scale (Hubbell et al. 1999). Typically, dominant species are prone to regeneration limitation, potentially leading to communities dominated by a single species during grassland development (Turnbull, Crawley, and Rees 2000). Some studies considered that plant population regeneration limitations should include seed, microhabitat and dispersal limitation (Muller‐Landau et al. 2002). Seed production quality and quantity determine subsequent processes such as germination and seedling establishment (Schupp, Milleron, and Russo 2002). Seed vigor, crucial for seed quality assessment, correlates closely with germination, seedling emergence, growth and stress tolerance (Al‐Amery et al. 2018). Microhabitat conditions, including light, soil moisture, nutrients and plant litter, significantly influence seed germination and seedling survival (Larpkern, Moe, and Totland 2011; Rotundo and Aguiar 2005). Furthermore, post‐maturation dispersal impacts seed and seedling access to light resources, excessive or inadequate light affects their survival on the QTP (Li and Ma 2003). Additionally, seeds and seedlings are vulnerable to feeding by cattle, sheep and other animals on the QTP, both of which ultimately affect plant population regeneration (Davidson, Detling, and Brown 2012). In summary, seed and microhabitat limitations are closely interconnected ecological processes profoundly affecting population regeneration, species composition and structure of community. Besides, we should also consider bud production and survival in population regeneration processes for clonal plants (Qian et al. 2017).

As a dioecious and clonal plant as well as a pioneer species in community succession, *Hippophae rhamnoides* plays an important role in soil and water conservation and biodiversity protection (Li et al. 2013). Recent years have seen a significant encroachment of *H. rhamnoides* in the alpine grassland, which often described as “a single tree becomes a forest, and should be immortal”. Despite this encroachment success, significant decline has occurred over the long‐term succession course following establishment on the QTP. Therefore, our research focuses on *H. rhamnoides* population regeneration, investigating their regeneration patterns and key limiting factors from two aspects: seed and microhabitat limitations. This approach aims to address the following scientific questions: (i) What regeneration strategies do *H. rhamnoides* populations adopt at different successional stages post‐encroachment, and what are the underlying mechanisms of influence? (ii) What are the key factors limiting the *H. rhamnoides* population regeneration? Our study aims to better predict the future developmental trajectory of *H. rhamnoides* populations and provide a theoretical foundation for managing the degradation and restoration of alpine grasslands impacted by shrub encroachment on the QTP.

## 2 Materials and Methods

### 2.1 Study Area

The study area is distributed across the eastern margin of the QTP near Hezuo city (33°06′30″–35°32′ N, 100°44′45″–104°45′ E) in Gansu province, China. This is a typical cold and humid type alpine region with a long cold season and a short warm season. The average annual temperature ranges from −0.5°C to 3.5°C, with an extreme maximum recorded of 28°C and an extreme minimum temperature of −23°C. The average annual precipitation is 545 mm, which is concentrated in the period from July to September. The altitude of the field sites is 2936 m, and there is a high diversity of vegetation.

### 2.2 Sample Selection and Investigation of Population Regeneration Patterns

We selected three successional stages representing early (34°57′07′′ N, 102 53′07′′ E), middle (34°57′58′′ N, 102°52′41″ E) and late (34°57′57′′ N, 102°52′27″ E) according to the average height and dead shoot rate of *H. rhamnoides* after encroachment. Seed production (SP, seeds/plant) was estimated using the proportion of whole fruit biomass from five plants at each succession in the winter of 2022. In May, 2023, five female plants were selected and total number of fruits in a single branch was used to estimate seed content of whole plant as canopy seed bank (CSB, seeds/plant). Three 10 × 10 m^2^ samples were selected to count the number of perennial seedlings (plants/m^2^) and ramets (produced from the horizontal roots of the mother plant by digging, plants/m), and three soil samples of 0–5, 5–10 and 10–20 cm soil layers were collected, brought back to the laboratory to wash out the soil transient seed bank (SSB, seeds/m^3^) (Fay and Olson 1978). In addition, five 50 × 50 cm^2^ sample areas were set up to count the number of one‐year seedlings (plants/m^2^). Plant litter was collected and the plant litter seed bank (PLSB, seeds/m^3^) was measured. And five plant horizontal roots were dug out using excavation method with the roots of the mother plant as starting point and below‐ground bud bank (BBB, bud/m^3^) was measured in May and August, 2023, respectively, using five sample plots of 50‐cm‐long, 30‐cm‐wide and 10‐cm‐deep, which ensure complete sampling to represent the belowground bud bank due to the horizontal root distribution was relatively shallow.

### 2.3 Determination of Soil Physical and Chemical Properties

A soil auger was used to collect soil samples at 0–20 cm depth using a five‐point sampling method. The soil was placed in aluminum boxes for determination of soil water content (SWC, %) and soil chemical properties. Soil total nitrogen (STN, g kg^−1^) was determined by indophenol blue spectrophotometry (Bremner and Mulvaney 1982). Soil total phosphorus (STP, g kg^−1^) content was determined by the molybdenum‐antimony anti‐colorimetric method (Wang et al. 2021). Soil organic carbon (SOC, g kg^−1^) was determined by the potassium dichromate oxidation method (Nóbrega et al. 2015), and soil pH was determined with a pH electrode. Soil fresh weight (m_1_, g) was recorded and samples dried at 105°C to record dry weight (m_2_, g).

(1) $$\mathrm{SWC}\;\left(\%\right)=\left(m_{1}-m_{2}\right)/m_{2}\times 100\%$$

(2) $$\mathrm{SBD}\;\left(g\;{\mathrm{cm}}^{-3}\right)=m/v$$

“*m*” is mass of dry soil in the ring knife, g; “*v*” is volume of ring knife, cm^3^.

### 2.4 Determination of Seed Emergence Traits

Seed length and width were determined with vernier calipers and seed length‐to‐width ratio (SLW) were calculated. In the laboratory, CSB, PLSB and SSB were weighed for seed hundred‐grain weight (SHW, g) with an electronic balance (0.0001 g). Seed coat thickness (SCT, mm) and water permeability (SCP, %) were measured by selecting some seeds at three successional stages, intact seeds were cut along the direction of the hilum with a scalpel to examine the palisade layer. Three seeds of *H. rhamnoides* at each succession were mounted on a stud, coated with gold and examined with a JSM‐5600LV (JEOL, Japan) scanning electron microscope at EHT = 5 kV, Mag = 200X. Seed vigor (SV) was determined with a electric conductivity meter by measuring the electric conductivity (EC, us cm^−1^) of seed soaking solution. Seed germination experiment setting CK, 20% NaOH, 40% NaOH, 60% NaOH and peeling treatments, 5 replicates per treatment, and 20 seeds per replicate. Germination was defined as successful if the emerging radicle reached a length of 2 mm. The number of seeds germinated, the germination initiation time (d), the germination period (d), and T_50_ (the time required for seed germination to reach 50%, d) until two consecutive days when there were no more new germinations were recorded and calculated:

(3) $$\text{Germination rate}\;\left(\%\right)=\frac{\text{number of normally germinated seeds}}{\text{total number of seeds}}\times 100\%$$

### 2.5 Simulating Shading and Feeding Treatments on *H. rhamnoides* Seedlings

In 2022, the light intensity under the forest at different successional stages was measured, and then we used the black shading nets of different thickness to simulate different light intensity for shading experiments on *H. rhamnoides* seedlings: two layers for moderate‐shading, and four layers for high shading. Leaves and branches were removed to simulate animal feeding (to simulate moderate feeding removed 50% of the leaves and some branches, and for high feeding we removed 80% of the leaves and most branches), while considering the interaction between two factors. The test seedlings while not ramets were selected for their robust and uniform growth, avoiding the effect of clonal integration to ramets, and the culture substrate was soil of alpine grassland. We set up nine treatments, which were non‐shading and feeding (NN), non‐shading and moderate‐feeding (NM), non‐shading and high‐feeding (NH), moderate‐shading and non‐feeding (MN), moderate‐shading and feeding (MM), moderate‐shading and high‐feeding (MH), high‐shading and non‐feeding (HN), high‐shading and moderate‐feeding (HM), high‐shading and feeding (HH). Five replications were set up for each treatment. All seedlings were subjected to the same care measures, and the position of pots was adjusted weekly, and the indexes were measured after 60 days.

Gas exchanges were measured from 9 to 11:30 a.m. on three consecutive cloudless and sunny days with a portable gas exchange and fluorescence GFS‐3000 system (WALZ, Effeltrich, Germany). Three seedlings were selected for each treatment, and light intensity was set to 1600 μmol m^−2^ s^−1^ (Fan et al. 2024). The leaves, roots and stems were separated in the laboratory, drying and weighing them at 80°C. The leaf biomass (LB, g), stem biomass (SB, g), root biomass (RB, g) and total biomass (TB, g) were determined with an electronic balance (0.01 g). We calculating the ratio of TB accounted for by each organ of the LMR, SMR and RMR. Leaf non‐structural carbon (NSC) was determined by the anthrone colorimetric method (Raessler et al. 2010).

### 2.6 Statistical Analyses

Excel was used for basic data sorting, and SPSS 26.0 was used for chi‐square test, single and two‐factor analysis of variance. A redundancy analysis (RDA) of soil properties on seed and bud traits of *H. rhamnoides* was performed with Canoco 5.0. The analytical results and principal components analysis (PCA) were plotted using Origin 2024, and the data in the graphs are means ± se. Structural equation modeling (SEM, R 4.3.0, piecewiseSEM) among soil factors, seed production, storage and seedlings regeneration was constructed the from the data after dimensionality reduction by PCA.

## 3 Results

### 3.1 Renewal Pattern of *H. rhamnoides* Population

**FIGURE 1:**
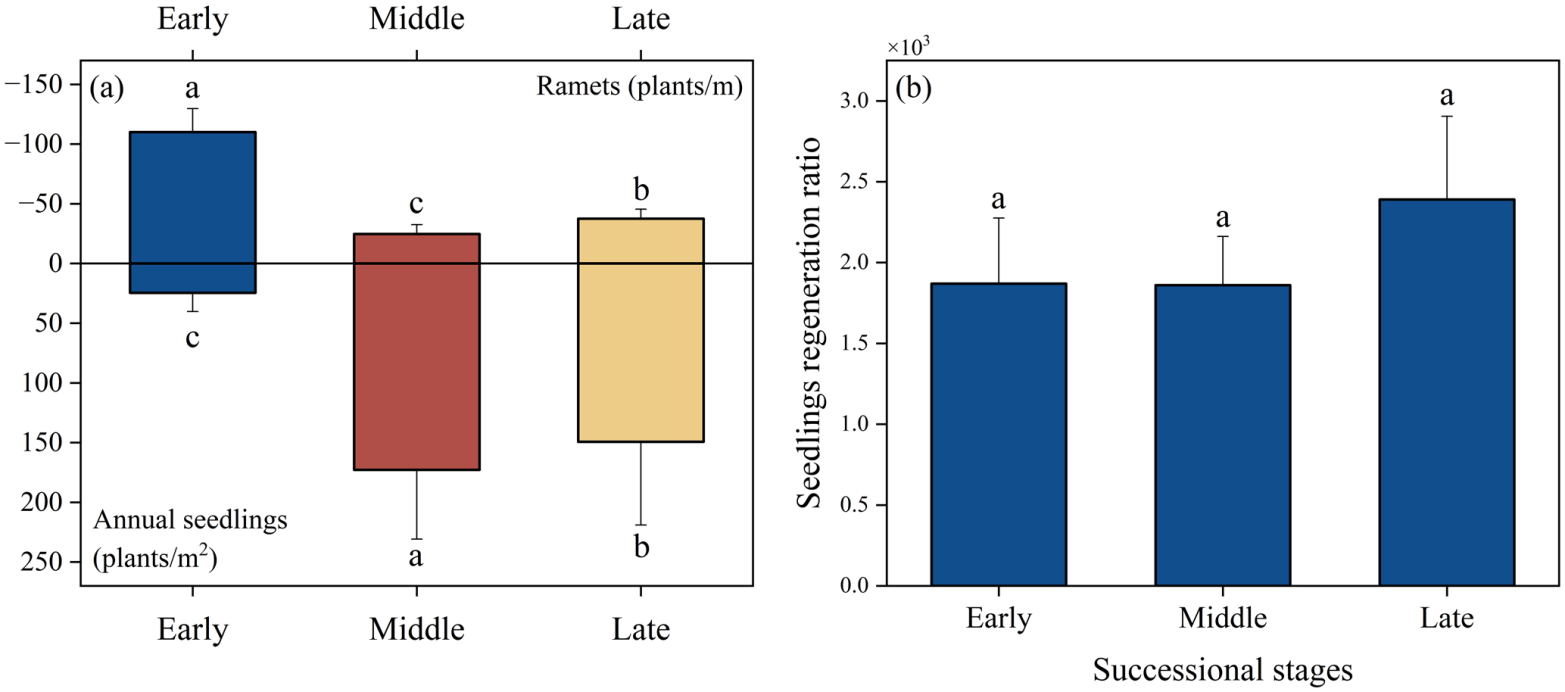
Regeneration pattern of *H. rhamnoides* population at three successional stages. (a) The density of annual seedlings and ramets at different successional stages. (b) The seedlings regeneration ratio at different successional stages. “a,” “b,” and “c” represent significant differences among the three successional stages.

The one‐year seedling was significantly lower in the early compared to other successional stages (*p* < 0.05). Conversely, the number of ramets in the early was significantly higher than in the middle and late (*p* < 0.05) (Figure 1). Additionally, the regeneration ratio of *H. rhamnoides* seedlings, whether through sexual and clonal reproduction, was observed to be low (Figure 1b).

### 3.2 Seed and Bud Limitation of *H. rhamnoides* Population Regeneration

#### 3.2.1 The Storage of *H. rhamnoides* Seed and Bud Bank at Different Successional Stages

The SP, SSB (0–5), SSB (5–10) and SSB (10–20) showed significant increases, with the highest SP observed in the late (*p* < 0.05). The CSB, PLSB, S‐BBB and A‐BBB exhibited an initial increase followed by a decrease with the succession, and significant differences in CSB were noted among the three successional stages (*p* < 0.05) (Table 1).

**TABLE 1:** The abundance of seed and bud bank across the three successional stages.

	SP (seeds/Plant)	CSB (seeds/Plant)	PLSB (seeds/m^3^)	SSB (seeds/m^3^)			BBB (buds/m^3^)	
				0–5 cm	5–10 cm	10–20 cm	Spring	Autumn
Early	5540.38 ± 661.61c	226.60 ± 44.65c	500.00 ± 152.78b	109.78 ± 41.86b	11.76 ± 7.84b	0.00 ± 0.00a	533.34 ± 117.38a	306.67 ± 85.89b
Middle	36,854.08 ± 2658.47b	3230.60 ± 711.37a	16,916.67 ± 4782.53a	523.43 ± 97.26b	117.62 ± 45.56a	8.82 ± 3.60a	640.00 ± 58.12a	1013.33 ± 245.31a
Late	72,748.70 ± 10,413.05a	1631.20 ± 305.81b	10,291.67 ± 1491.91a	1666.36 ± 275.37a	160.75 ± 53.11a	217.61 ± 168.05a	586.67 ± 90.43a	680.00 ± 197.09a

*Note:* “a,” “b,” and “c” represent significant differences among the three successional stages.

Abbreviations: BBB, below‐ground bud bank; CSB, canopy seed bank; PLSB, plant litter seed bank; SP, seed production; SSB, soil seed bank.

#### 3.2.2 Seed Morphological Characteristics at Different Successional Stages

*H. rhamnoides* seeds are purple‐black in color, with a glossy surface and a broadly elliptic to ovate shape. The SLW initially decreased and then increased, but this trend was not significant among the three successional stages. Conversely, within the same seed bank, higher SHW values were consistently observed in the middle. Specifically, SHW‐CSB was higher than SHW‐PLSB and SHW‐SSB in the early, while SHW‐PLSB exceeded SHW‐CSB and SHW‐SSB in the middle and late. Additionally, higher SCT and SCP were evident in the middle, with SCT showing significant differences among the three successional stages (*p* < 0.05), while SCP did not (Table 2).

**TABLE 2:** Seed characteristics at the three successional stages.

Successional stages	SLW	SHW (g)			SCT (mm)	SCP (%)
		CSB	PLSB	SSB (0–5 cm)		
Early	1.37 ± 0.02a	0.86 ± 0.04a	0.84 ± 0.04b	0.75 ± 0.03b	0.77 ± 0.02b	3.94 ± 1.86a
Middle	1.28 ± 0.03a	0.91 ± 0.06a	1.09 ± 0.10a	0.99 ± 0.09a	0.81 ± 0.02a	9.13 ± 4.69a
Late	1.37 ± 0.09a	0.69 ± 0.04b	0.75 ± 0.06b	0.68 ± 0.03b	0.33 ± 0.01c	7.24 ± 2.76a

*Note:* “a,” “b,” and “c” represent significant differences among the three successional stages.

Abbreviations: SCP, seed coat water permeability. SCT, seed coat thickness; SHW, seed hundred‐grain weight; SLW, seed length to width ratio.

#### 3.2.3 Seed Vigor of *H. rhamnoides* at Different Successional Stages

**FIGURE 2:**
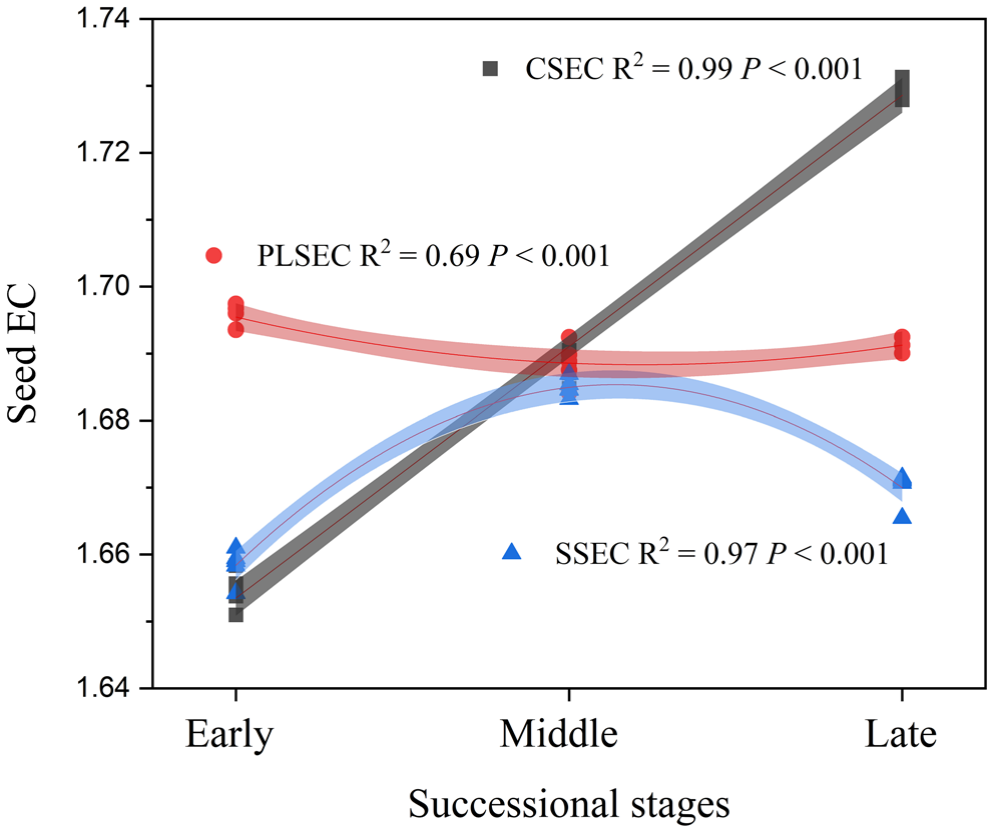
The electric conductivity of *H. rhamnoides* seeds soaking solution at different successional stages.

The results of seed soaking solution experiment revealed that SV‐CSB decreased significantly, SV‐PLSB increased, and SV‐SSB initially decreased and then increased with the succession. Over time, SV‐CSB and SV‐SSB showed a gradual increase, whereas SV‐PLSB exhibited the opposite trend. In early succession seeds, SV‐CSB and SV‐SSB were significantly higher than SV‐PLSB. However, SV‐CSB, SV‐PLSB, and SV‐SSB gradually decreased in middle and late succession seeds. In late succession seeds, SV‐SSB>SV‐PLSB>SV‐CSB, with no significant differences among the three seed banks observed in middle succession seeds (Figures 2 and S1).

**FIGURE 3:**
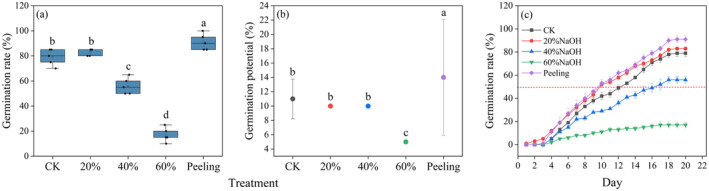
Effects of different treatments on germination rate and germination process of *H. rhamnoides* . (a) Seed germination rate under different treatments. (b) Germination potential under different treatments. (c) Seed germination process under different treatments. “a,” “b,” “c,” and “d” represent significant differences among different treatments.

**FIGURE 4:**
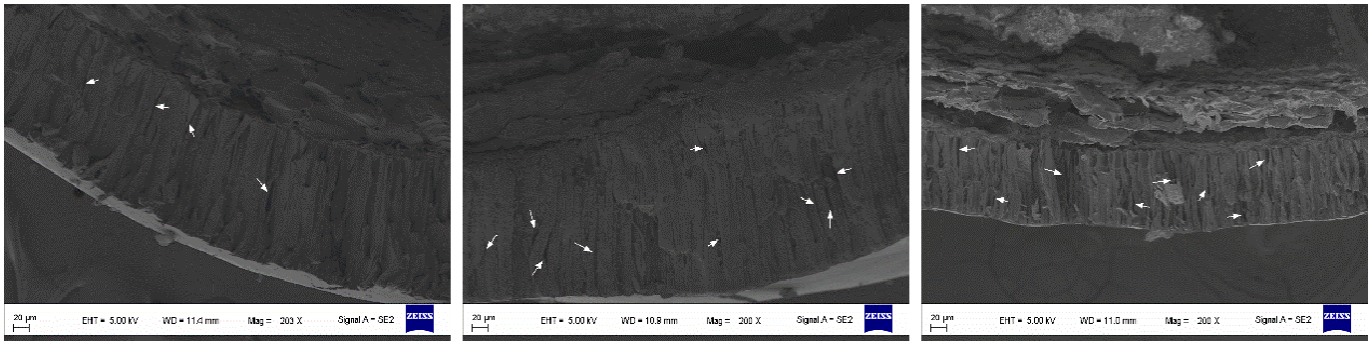
Seed anatomical structure of *H. rhamnoides* at different successional stages. The anatomical structure of early, middle, and late seed‐coat was observed from left to right, and the white arrow represents the seed coat palisade gap.

With respect to seed germination, the results indicated that treatment with 20% NaOH increased the germination rate by approximately 4% and reduced T_50_. Conversely, treatments with 40% and 60% NaOH significantly decreased the germination rate about 23% and 62%, respectively. Peeling treatment increased the germination rate by approximately 12% and also shortened T_50_. Anatomical examination revealed conspicuous “gaps” in the seed coat palisade layer across all successional stages, with a higher density of these gaps observed in the middle compared to the early and late (Figures 3 and 4).

#### 3.2.4 PCA Of *H. rhamnoides* Population Regeneration

**FIGURE 5:**
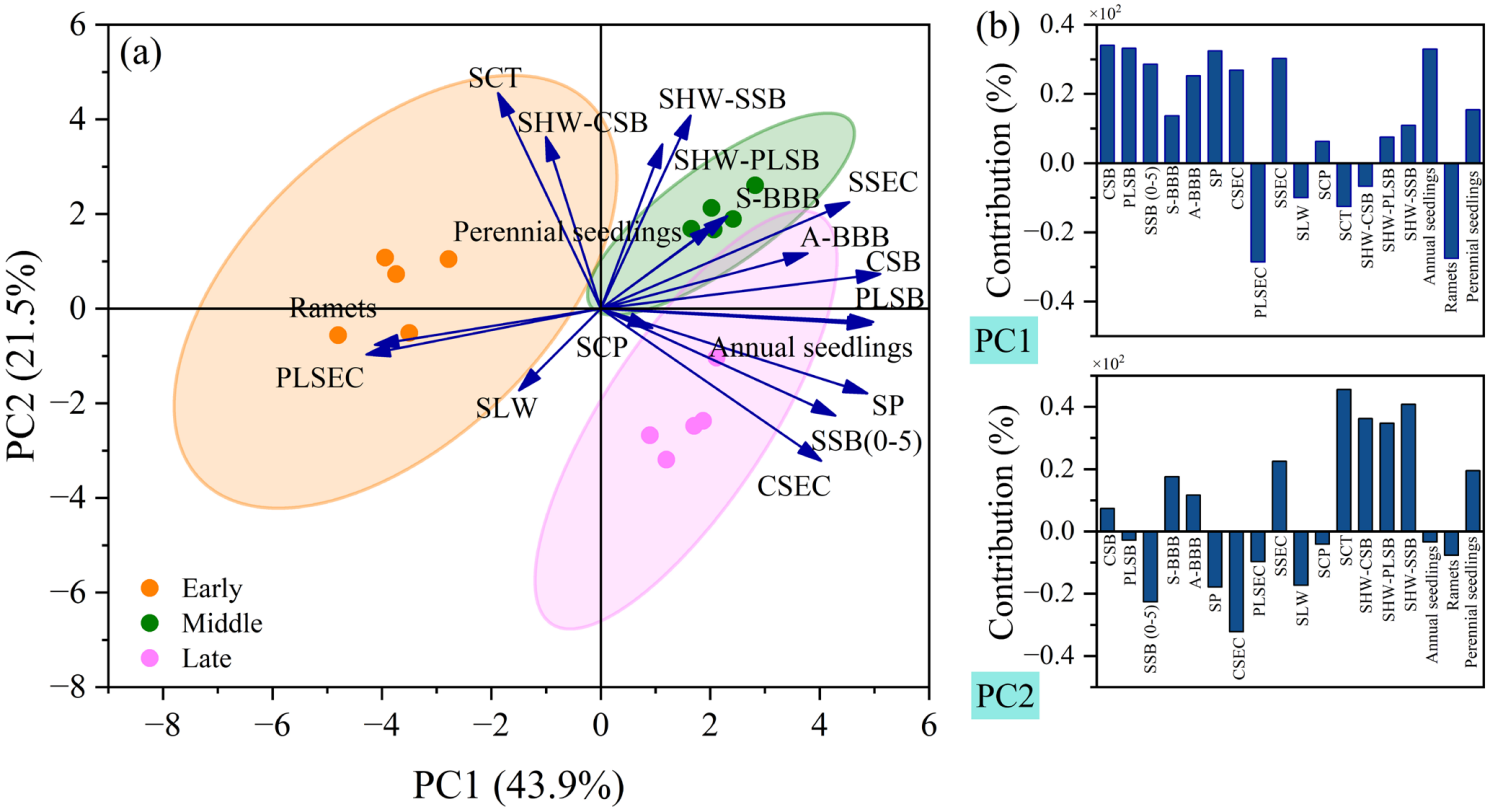
PCA of *H. rhamnoides* population regeneration traits. (a) PCA of *H. rhamnoides* population regeneration traits. (b) The contribution of regeneration traits to PC1 and PC2 axis. A‐BBB, below‐ground bud bank in Autumn; CSB, canopy seed bank; CSEC, the electric conductivity of canopy seeds; PLSB, plant litter seed bank; PLSEC, the electric conductivity of plant litter seeds; S‐BBB, below‐ground bud bank in Spring; SCP, seed coat water permeability; SCT, seed coat thickness; SHW‐CSB, seed hundred‐grain weight of canopy seeds; SHW‐PLSB, seed hundred‐grain weight of plant litter seeds; SHW‐SSB, seed hundred‐grain weight of soil seeds; SLW, seed length to width ratio; SP, seed production; SSB, soil seed bank; SSEC, the electric conductivity of soil seeds.

Overall, PCA revealed that the first two principal components explained 43.9% and 21.5% of the variation in *H. rhamnoides populations* regeneration traits. The findings indicated that annual seedling regeneration exhibited a significant positive correlation with CSB, PLSB and SSB. In contrast, perennial seedling regeneration showed a significant positive correlation with A‐BBB and S‐BBB. Additionally, ramet regeneration displayed a negative correlation with SSB and BBB (Figure 5).

### 3.3 Microsite Limitation of *H. rhamnoides* Population Regeneration

#### 3.3.1 Effect of Soil Physico‐Chemical Properties on Seed Traits

**FIGURE 6:**
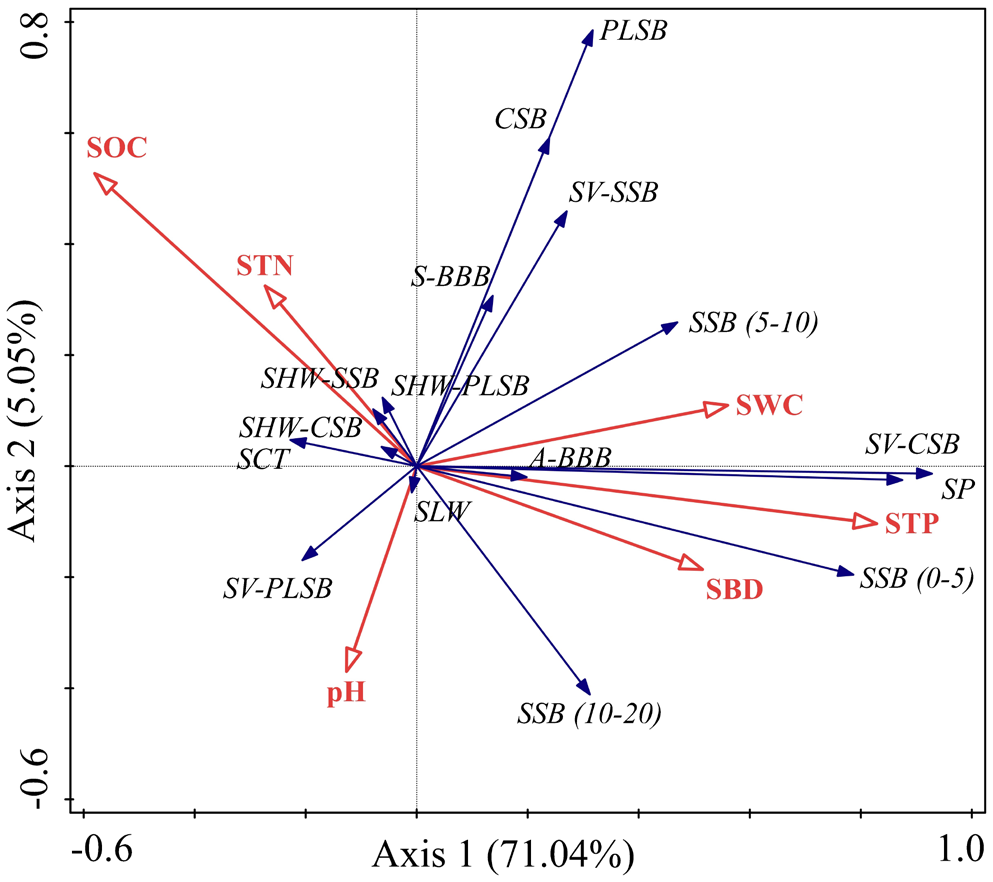
Effects of soil properties on *H. rhamnoides* population regeneration traits. (a) RDA of soil factors on regeneration traits of *H. rhamnoides* . (b) The path analysis of *H. rhamnoides* population regeneration by STP, and red arrow indicates a positive correlation, blue arrow indicates a negative correlation, dotted lines indicate insignificant, and thicker solid lines indicate stronger correlations. *R* ^2^ next to the endogenous variables indicate their explained variance, *p < 0.05; **p < 0.01. SV‐CSB, The seed vigor of canopy seeds; SV‐PLSB, The seed vigor of plant litter seeds; SV‐SSB, the full names of other indicators are shown in the legends of Figure 5.

The SWC, SBD and STP (*p* < 0.05) exhibited a clear increasing trend as succession progressed. In contrast, SOC sharply decreased with the succession (*p* < 0.05), while STN initially increased and then decreased. Soil pH showed a marginal decrease across the three successional stages (Table S1). The first two axes of RDA explained total variations of 76.09%. STP significantly influenced the variation in *H. rhamnoides* seed and bud traits (*p* < 0.001). SWC, SBD and STP had a stronger effect on SP, A‐BBB, SSB (0–5) and SV‐CSB. SOC and STN had a greater impact on SHW and SCT across the three seed banks, while pH had a greater effect on SV‐PLSB, SLW and SSB (10–20) (Figure 6).

#### 3.3.2 Effects of Shading and Feeding on the Survival Rate of *H. rhamnoides* Seedlings

The *X* ^2^ test results showed that single feeding had no significant effect, while single shading treatment had a significant effect on the survival rate of *H. rhamnoides* seedlings (*p* < 0.05). Under the interaction of shading and feeding, MM, MH, HM and HH treatments can significantly affect the survival rate of seedlings (*p* < 0.01) (Table 3).

**TABLE 3:** Effects of shading and feeding on the survival rate of *H. rhamnoides* seedlings.

Treatment	Pearson *X* ^2^ test		
	*X* ^2^ value	df	Asymptotic significance (2‐sided)
Feeding	3.333	2	0.178
Shading	5.455	2	0.020
Shading × Feeding	11.806	4	0.005

#### 3.3.3 Effects of Shading and Feeding on the Growth of *H. rhamnoides* Seedlings

The results indicated that MN significantly increased seedling Pn, while MM and HM significantly reduced it (*p* < 0.05). However, HN and NM showed no significant effect on Pn (similar to NN). Meanwhile, MN, NM and MM improved leaf NSC compared to NN, whereas HN and HM reduced it. As shading intensity increased, SMR also increased, while RMR significantly decreased compared to NN. MN increased LMR, whereas HN decreased it. Two‐factor analysis of variance revealed that both photosynthetic rate and biomass accumulation of seedlings were significantly influenced by single shading or feeding factors as well as their interaction (Table 4).

**TABLE 4:** Effects of shading and feeding on seedling growth of *H. rhamnoides* (two factors).

Variable	df	Pn		Soluble sugar		Starch		NSC		Sugar starch ratio	
		*F*	*P*	*F*	*P*	*F*	*P*	*F*	*P*	*F*	*P*
Shading	2	31.23	0	248.45	0	22.47	0	205.53	0	23.80	0
Feeding	1	70.50	0	2.58	0.12	16.35	0	6.47	0.02	20.03	0
Shading × Feeding	2	21.73	0	94.50	0	21.63	0	124.99	0	1.51	0.24

## 4 Discussion

### 4.1 Population Regeneration Strategies After Shrub Encroachment

Plant population regeneration starting with seed production followed by dispersal, storage, germination, seedlings establishment and growth, and finally the recruitment of seedlings that exceed a certain measurement threshold under the influence of various biotic and abiotic factors (de Carvalho et al. 2017). Concurrently, clonal reproduction also plays an important ecological role in population regeneration (Liu, Liu, and Dong 2016). Our results revealed that seed production (SP) was significantly lower in the early compared to middle and late. Below‐ground bud bank in Autumn (A‐BBB) was lower than below‐ground bud bank in Spring (S‐BBB) in the early, and significantly lower than middle and late. *H. rhamnoides* population had more ramets in the early, while there was a higher density of one‐year seedlings from sexual reproduction in the middle and late, indicating a trade‐off between sexual and clonal reproduction. This shift from predominant clonal reproduction in the early to higher sexual reproduction in the middle and late likely enhances genetic diversity as environmental conditions improve with the succession (Table S1). Sexual reproduction is particularly advantageous for colonizing new environments and establishing new populations (Eriksson 1997), whereas clonal reproduction becomes more prevalent after initial seed establishment, allowing rapid occupation of favorable spaces with fewer constraints (Pérez‐Harguindeguy et al. 2013). The trade‐off between bud and seed banks may represent complementary strategies to buffer against the effects of climate change in grassland ecosystems (Vandvik and Goldberg 2006). Furthermore, our results highlighted that STP had the most significant impact on population regeneration strategies and seedling emergence traits of *H. rhamnoides* . This influence may stem from phosphorus limitation in grassland ecosystems, exacerbated by overgrazing and limited artificial fertilization and management practices (Sitters, Cherif, and Egelkraut 2019). STP affects seed production and vigor, thereby influencing seedling and seed bank regeneration, and ultimately shaping *H. rhamnoides* population dynamics (Figure 6). As phosphorus gradually supplants nitrogen as the primary limiting factor for plant growth and ecosystem functioning, its availability becomes crucial (Tahovská et al. 2018).

Additionally, our study revealed a low regeneration ratio of seedlings through both sexual and clonal reproduction pathways, underscoring limitation on population regeneration. According to theories of population regeneration limitation, plants face limitation at various stages, including seed, dispersal, and microhabitat (Muller‐Landau et al. 2002). Ensuring an adequate supply of viable seeds and successful establishment in suitable microhabitats are critical steps determining the success of population regeneration. Failures at any stage can lead to poor population regeneration outcomes (Maron et al. 2019).

### 4.2 Limiting Factors for Population Regeneration and Recruitment

#### 4.2.1 Seed and Bud Limitation on *H. rhamnoides* Population Regeneration

After dispersal from the parent plant, seeds reach the ground via seed rain and either germinate immediately, form a short‐lived or persistent seed bank, delaying their renewal (Plue and Cousins 2013). Seed rain is a crucial stage linking plant reproduction to subsequent life history phases (Nathan and Muller‐Landau 2000). Seeds will be affected by various biotic and abiotic factors, most die in the diffusion and only a few seeds can successfully germinate, build seedlings and complete the renewal process (Frei et al. 2018). Our results indicated that canopy seed bank (CSB) and plant litter seed bank (PLSB) initially increased and then declined with the succession, possibly due to the rapid demise of annual or perennial grasses with shallow roots under competitive pressure for nutrients and water in the local environment, as posited by the “resource pool hypothesis” (Ryel et al. 2008). Wind effects, particularly in larger understory gaps, likely contribute to lower CSB and PLSB in the early and late compared to the middle (Table 1). Moreover, soil seed bank (SSB) was significantly higher in the late than in the early and middle (*p* < 0.05), showing a linear increase with the succession, consistent with previous studies (Kalamees et al. 2012; Ma et al. 2010, 2018). Additionally, we observed that S‐BBB predominated over A‐BBB in the early, while A‐BBB surpassed S‐BBB in the middle and late. Both types of *H. rhamnoides* bud banks initially increased and then decreased with succession. Our results indicate a significant decrease in bud banks alongside an increase in ramets, confirming adjustments in *H. rhamnoides* regeneration strategies following seed dispersal.

Seed vigor (SV) determines the potential for rapid and uniform emergence and development under diverse field conditions (Rajjou et al. 2012). Our results found that SV‐CSB and SSB were more vigorous than PLSB, suggesting that *H. rhamnoides* seedlings mainly derive from CSB and SSB in the early. Seed vigor was higher across all seed banks in the middle, with SV‐PLSB and SV‐SSB significantly exceeding that of CSB in the late, serving as direct regeneration sources. However, SV‐CSB exhibited a decreasing trend with the succession, potentially due to extensive dieback in the lower and middle parts of aging *H. rhamnoides* . CSB plays a role analogous to SSB in enabling plants to withstand adverse external conditions (Lamont et al. 1991). The increased SV‐PLSB may stem from enhanced plant litter cover, aiding in moisture retention as succession progressed (Rotundo and Aguiar 2005). Moreover, our study indicated that natural seed germination in *H. rhamnoides* reached 80%, with peeling treatment significantly enhancing germination rates and reducing T_50_, highlighting the presence of physical dormancy due to the seed coat's mechanical effects in previous studies (Harrison and Beveridge 2002). The thickness and presence of the seed coat not only affect water and air permeability but also mechanically inhibit embryo growth (Willis et al. 2014). We found that permeability did not vary with changes in thickness of seed coat across the succession, with the highest permeability and thickness of seed coat observed in the middle. Physical dormancy arises from a water‐impermeable palisade layer within the seed coat, which must become permeable for water passage to enable seed germination (Baskin and Baskin 1998; Fang et al. 2017). Our results revealed conspicuous gaps in the palisade layer of *H. rhamnoides* seed coats across different successional stages, suggesting these gaps may function as “water transport channels,” alleviating mechanical barriers imposed by thicker seed coats and contributing to higher natural germination rates.

#### 4.2.2 Microhabitat Limitation on *H. rhamnoides* Population Regeneration

A suitable habitat comprises external conditions essential for seedling regeneration (Grubb 1977). Microhabitat limitations primarily affect seed germination and seedling survival, influenced by light, water, temperature, and nutrients (Dupuy and Chazdon 2008). Soil properties, including pH and nutrient levels, are critical in shaping the soil seed bank (Roem, Klees, and Berendse 2002; Zhao et al. 2021). Our results indicated that pH primarily influenced SV‐PLSB and SSB, potentially by mitigating pathogenic fungi (Basto et al. 2013). Studies have shown that higher pH correlates with lower soil seed density on the QTP, consistent with our findings (Ma et al. 2017). SWC, SBD and STP exerted significant effects on seed production, A‐BBB and SSB. Previous research suggested that bud density varies with increasing SWC, extreme drought can compromise seed physiological mechanisms, thereby reducing seed vigor (Kranner et al. 2010). Additionally, SBD has been shown to predict soil seed density, increasing and stabilizing with higher SBD, which aligns with our finding of a positive correlation between SBD and SSB (Yang et al. 2021). The role of SSB is crucial, synergizing with seed rain to promote *H. rhamnoides* population regeneration.

Seedling establishment represents a vulnerable stage in plant life history, susceptible to significant losses (Rees et al. 2001; Moles and Westoby 2004). Environmental factors and animal feeding are major contributors to seedling mortality (Moles and Westoby 2004). Light availability strongly influences seedling growth; inadequate light or higher UV radiation can impair growth and survival during community establishment (Hérault and Hiernaux 2004; Scotto et al. 1988). Feeding is another biotic factor affecting seed germination and seedling growth (Moles and Westoby 2004). Our results indicated that shading significantly reduced *H. rhamnoides* seedling survival compared to feeding alone (*p* < 0.05), with a significant interaction effect (*p* < 0.01). Moreover, successful seedling establishment and recruitment depend on biomass allocation patterns and growth plasticity (Rees et al. 2001; Miner et al. 2005). Our findings showed that MN significantly increased seedling Pn and investment in leaf, with increased investment in root as shading intensified, which suggested seedlings would allocate more resources to functional organs to optimize resource acquisition, a strategy supported by biomass allocation theory (Müller, Schmid, and Weiner 2000). Numerous studies indicate that moderate shading benefits woody plant seedling growth and biomass accumulation (Deguchi and Koyama 2020; Sefcik, Zak, and Ellsworth 2006).

NSC derived from photosynthesis provide energy for plant metabolism, growth, and stress responses (Hoch, Richter, and Körner 2003; Hartmann and Trumbore 2016). MM and HM significantly decreased Pn, with MM increasing NSC and the sugar–starch ratio while HM decreased them. Our results also demonstrated that NSC and the sugar–starch ratio in seedlings initially increased and then decreased with increasing shading levels. NM treatment increased NSC but decreased the sugar–starch ratio. These findings suggested shading directly limits photosynthesis, altering carbon assimilation and NSC dynamics. The sugar–starch system adjusts to environmental changes by regulating soluble sugar–starch interconversion (Li et al. 2008; Han et al. 2020). Moreover, most of the sunny species typically adopt a “higher growth, lower storage” strategy, favoring above‐ground growth to escape shading conditions, maintaining a high‐soluble sugar to starch ratio to enhance water regulation and efficiency (Du et al. 2020; Sala, Woodruff, and Meinzer 2012). This aligns with our findings, highlighting the adaptive responses of plants to light availability and their carbohydrate metabolism under varying environmental conditions.

## 5 Conclusions

After *H. rhamnoides* encroached into alpine meadows, there were sufficient SP, CSB, PLSB, SSB, and BBB, with high seed vigor and seedling emergence. *H. rhamnoides* population regeneration is minimally limited by the quantity or quality of seed and bud. The dynamic effectiveness of STP is the primary mechanism influencing *H. rhamnoides* population regeneration. Light availability and feeding are identified as the key microhabitat factors restricting *H. rhamnoides* population regeneration.

## Author Contributions

**Baoli Fan:** conceptualization (lead), funding acquisition (lead), investigation (lead), methodology (lead), project administration (lead), resources (lead), supervision (supporting), writing – original draft (supporting), writing – review and editing (supporting). **Pengfei Gao:** conceptualization (lead), data curation (lead), formal analysis (lead), investigation (lead), methodology (lead), resources (lead), software (lead), supervision (lead), validation (lead), visualization (lead), writing – original draft (lead), writing – review and editing (lead). **Tingting Tian:** data curation (supporting), investigation (supporting), resources (supporting), visualization (supporting). **Jinhua Jiang:** data curation (supporting), investigation (supporting), resources (supporting), visualization (supporting). **Nana Ding:** data curation (supporting), investigation (supporting), resources (supporting). **Yongkuan Wan:** data curation (supporting), investigation (supporting). **Miaojun Ma:** methodology (supporting), resources (supporting), visualization (supporting). **Kun Sun:** conceptualization (supporting), methodology (supporting), resources (supporting).

## Conflicts of Interest

The authors declare no conflicts of interest.

## Supporting information


Data S1.

## Data Availability

The data that support the findings of this study are openly available in figshare at https://figshare.com/s/f4b4243356b88bdbb91b.
