# Supplementary material for: Regeneration Limitations of Hippophae rhamnoides Population After Successfully Encroached on the Qinghai‐Tibetan Plateau
Source: Ecol Evol. 2024 Dec 22;14(12):e70684. doi: 10.1002/ece3.70684 (PMC11663628; doi:10.1002/ece3.70684)

**Supplementary material**

**Table S1:** Characteristic values of soil environmental factors at three successional stages. “a”, “b” and “c” represent significant differences among the three successional stages.

| Successional stages | SWC  (%) | SBD  (g cm^-3^) | STP  (g Kg^-1^) | STN  (g Kg^-1^) | pH | SOC  (g Kg^-1^) |
| --- | --- | --- | --- | --- | --- | --- |
| Early | 19.10 ± 2.20a | 1.02 ± 0.08a | 0.27 ± 0.01c | 0.84 ± 0.21a | 7.57 ± 0.09a | 75.43 ± 10.58a |
| Middle | 20.63 ± 2.44a | 1.06 ± 0.06a | 0.31 ± 0.02b | 1.06 ± 0.14a | 7.53 ± 0.07a | 73.59 ± 15.84b |
| Late | 24.48 ± 1.52a | 1.19 ± 0.04a | 0.38 ± 0.01a | 0.60 ± 0.09a | 7.50 ± 0.09a | 28.92 ± 7.66c |

**Figure S1:** The electric conductivity of seed soaking solution at three successional stages. (a) The electric conductivity of seed soaking solution in the early at different types of seed banks. (b) The electric conductivity of seed soaking solution in the middle at different types of seed banks. (c) The electric conductivity of seed soaking solution in the late at different types of seed banks.


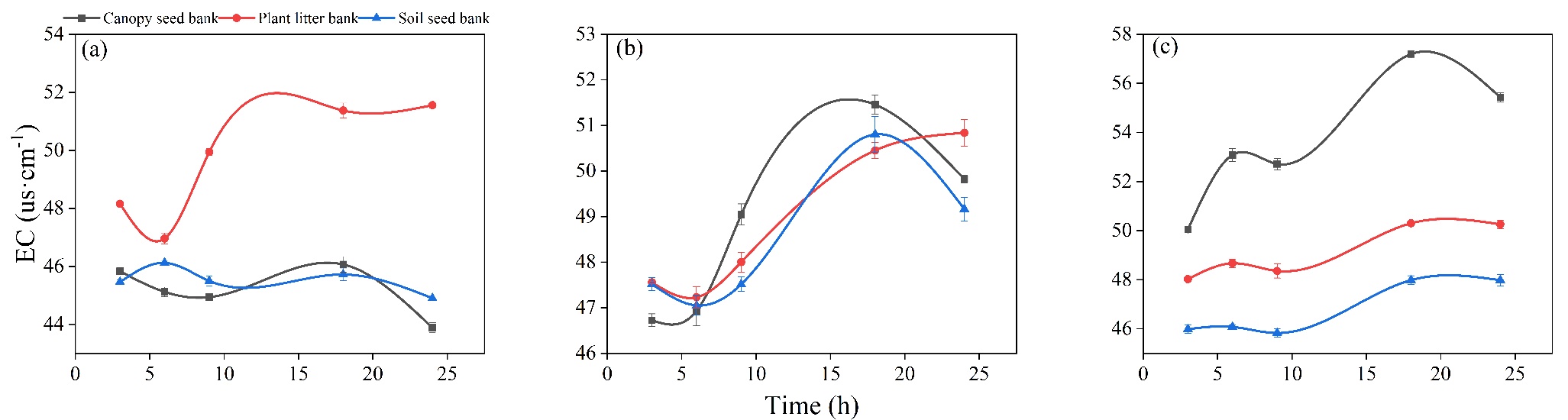

Supplement: Supplementary file 1 — Data S1. [file ECE3-14-e70684-s001.docx]
